# Dynamic Expansion and Contraction of *cagA* Copy Number in *Helicobacter pylori* Impact Development of Gastric Disease

**DOI:** 10.1128/mBio.01779-16

**Published:** 2017-02-21

**Authors:** Sungil Jang, Hanfu Su, Faith C. Blum, Sarang Bae, Yun Hui Choi, Aeryun Kim, Youngmin A. Hong, Jinmoon Kim, Ji-Hye Kim, Niluka Gunawardhana, Yeong-Eui Jeon, Yun-Jung Yoo, D. Scott Merrell, Linhu Ge, Jeong-Heon Cha

**Affiliations:** a Department of Oral Biology, Oral Science Research Center, Department of Applied Life Science, The Graduate School, BK21 Plus Project, Yonsei University College of Dentistry, Seoul, Republic of Korea; b Department of Microbiology and Immunology, Uniformed Services University of the Health Sciences, Bethesda, Maryland, USA; c Department of Dental Hygiene, Jeonju Kijeon College, Jeonju, Republic of Korea; d Microbiology & Molecular Biology, Key Laboratory of Oral Medicine, Guangzhou Institute of Oral Disease, Stomatology Hospital of Guangzhou Medical University, China; New York University

## Abstract

Infection with *Helicobacter pylori* is a major risk factor for development of gastric disease, including gastric cancer. Patients infected with *H. pylori* strains that express CagA are at even greater risk of gastric carcinoma. Given the importance of CagA, this report describes a new molecular mechanism by which the *cagA* copy number dynamically expands and contracts in *H. pylori*. Analysis of strain PMSS1 revealed a heterogeneous population in terms of numbers of *cagA* copies; strains carried from zero to four copies of *cagA* that were arranged as direct repeats within the chromosome. Each of the multiple copies of *cagA* was expressed and encoded functional CagA; strains with more *cagA* repeats exhibited higher levels of CagA expression and increased levels of delivery and phosphorylation of CagA within host cells. This concomitantly resulted in more virulent phenotypes as measured by cell elongation and interleukin-8 (IL-8) induction. Sequence analysis of the repeat region revealed three *cagA* homologous areas (CHAs) within the *cagA* repeats. Of these, CHA-ud flanked each of the *cagA* copies and is likely important for the dynamic variation of *cagA* copy numbers. Analysis of a large panel of clinical isolates showed that 7.5% of *H. pylori* strains isolated in the United States harbored multiple *cagA* repeats, while none of the tested Korean isolates carried more than one copy of *cagA*. Finally, *H. pylori* strains carrying multiple *cagA* copies were differentially associated with gastric disease. Thus, the dynamic expansion and contraction of *cagA* copy numbers may serve as a novel mechanism by which *H. pylori* modulates gastric disease development.

## IMPORTANCE

Severity of *H. pylori*-associated disease is directly associated with carriage of the CagA toxin. Though the sequences of the CagA protein can differ across strains, previous analyses showed that virtually all *H. pylori* strains carry one or no copies of *cagA*. This study showed that *H. pylori* can carry multiple tandem copies of *cagA* that can change dynamically. Isolates harboring more *cagA* copies produced more CagA, thus enhancing toxicity to host cells. Analysis of 314 *H. pylori* clinical strains isolated from patients in South Korea and the United States showed that 7.5% of clinical strains in the United States carried multiple *cagA* copies whereas none of the South Korean strains did. This study demonstrated a novel molecular mechanism by which *H. pylori* dynamically modulates *cagA* copy number, which affects CagA expression and activity and may impact downstream development of gastric disease.

## INTRODUCTION

*Helicobacter pylori* is a Gram-negative microaerophilic bacterium that colonizes the human stomach and infects more than half of the world’s population (1–3). *H. pylori* infection is associated with various gastric diseases, ranging from gastritis to gastric adenocarcinoma and mucosa-associated lymphoid tissue (MALT) lymphoma (4, 5); the latter associations led the International Agency for Research on Cancer to classify *H. pylori* as a group I carcinogen (6). The high infection rates seen with this bacterium are believed to be responsible for making gastric cancer the third most common cause of cancer-related death worldwide (7).

*H. pylori* produces many virulence factors that contribute to pathogenesis (8, 9). Of these factors, cytotoxin-associated gene A (CagA) is one of the most widely studied proteins because of its association with increased risk of development of severe gastric diseases (10, 11). The *cagA* gene is carried on the *cag* pathogenicity island (PAI), which encodes a type IV secretion system (T4SS) that directly injects CagA into host cells (12). Once inside the host cell, CagA is phosphorylated by host cell kinases, forms a complex with SHP-2 (Src homology region 2-containing phosphatase 2) (13), and alters multiple host signaling pathways (13–17). Phosphorylation of CagA occurs in the carboxyl terminus on the conserved tyrosine residue within a repeated five-amino-acid sequence, Glu-Pro-Ile-Tyr-Ala, referred to as the EPIYA motif (13, 15). Both phosphorylated and nonphosphorylated forms of CagA modulate host cellular signaling pathways (18–20). The numbers of EPIYA motifs and the flanking regions surrounding these motifs differ dramatically across strains, making CagA highly polymorphic. On the basis of surrounding amino acid sequences, four distinct EPIYA motifs have been identified: EPIYA-A, EPIYA-B, EPIYA-C, and EPIYA-D. Interestingly, the distributions of EPIYA motif combinations differ geographically (15, 21); East Asian strains contain EPIYA-ABD, whereas Western strains contain EPIYA-ABC, and the EPIYA-C motif may be repeated up to five times (13, 15, 22). These different EPIYA combinations have been shown to have an impact on disease progression (21, 23). Indeed, our prior study showed a significant association between infection with *H. pylori* strains carrying the EPIYA-ABD *cagA* genotype and the development of gastric cancer (23). Similarly, other studies have shown that CagA variants containing an increased number of EPIYA-C motifs correlate to more virulent disease characteristics (15, 24, 25).

In addition to toxin amino acid variation, the *cagA* promoter region has also been shown to be genetically heterogeneous; an AATAAGATA motif located +59 bp upstream of the transcription start site is associated with higher levels of CagA expression in *H. pylori* isolates from Colombia (26, 27) and Portugal (28). These increased levels of CagA result in higher levels of interleukin-8 (IL-8) secretion by gastric cells *in vitro*. In populations at high risk for gastric carcinoma, strains containing the +59 motif are associated with more advanced precancerous lesions (27) and intestinal metaplasia (28). Thus, the amount of CagA expressed by *H. pylori* appears to be linked to downstream pathogenesis.

The host signaling pathways that are modified by CagA upon translocation into host cells stimulate cytoskeletal rearrangement, increased cellular mobility, and elongated cell shape, referred to as the “hummingbird phenotype” (14, 29). Additionally, *H. pylori* induces secretion of the proinflammatory cytokine IL-8 from gastric epithelial cells by a T4SS-dependent mechanism (30–33). The T4SS apparatus itself induces IL-8 secretion during the early phase of infection, and CagA augments IL-8 secretion in later phases (34).

As a bacterial species, *H. pylori* shows exceptionally high rates of genetic variability and intraspecies diversity (35–39). It is believed that these genetic differences likely influence the overall virulence potential of individual strains. Among the variable factors, the members of the outer membrane protein (OMP) families, such as adherence-associated lipoprotein A and B (AlpA and AlpB), blood group antigen binding adhesin (BabA), *Helicobacter* outer membrane B (HomB), *Helicobacter* outer membrane protein Z (HopZ), outer membrane inflammatory protein A (OipA), and sialic acid binding adhesin (SabA), show high genetic variability on the basis of the presence or absence of different closely related paralogs (40–46). For example, the *bab* family is made up of three paralogs (*babA*, *babB*, and *babC*) that can be located at three different chromosomal loci, referred to as locus A, locus B, and locus C (47–49). The notable exception is *oipA*, because duplicated *oipA* genes, not paralogs, may be located at two different loci (50). This DNA duplication event is thought to be mechanistically associated with DNA inversion (51).

Here, we describe a novel molecular mechanism by which *H. pylori* alters *cagA* copy number by dynamic expansion and contraction of *cagA* at the PAI locus. These changes in *cagA* copy number affect CagA expression; strains carrying multiple copies produce more toxin, which results in increased host cell elongation and IL-8 secretion. Thus, this mechanism of *cagA* variation promotes adaptation and pathogenesis of *H. pylori*.

## RESULTS

### *H. pylori* strain PMSS1 carries multiple *cagA* copies.

**FIG 1:**
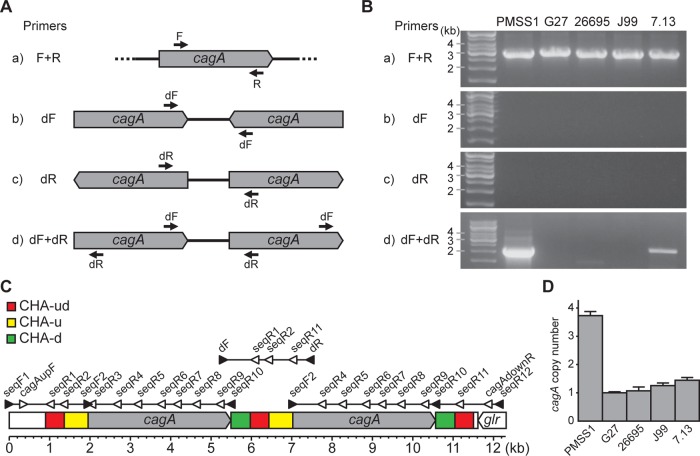
Analysis of *cagA* copy number by PCR and real-time PCR. (A) A scheme for the PCR-based method designed to detect multiple *cagA* genes and to identify the gene orientation. Primer annealing sites are shown. (B) *H. pylori* strains PMSS1, G27, 26695, J99, and 7.13 were analyzed for the presence of multiple copies of *cagA* using the four PCR sets. (C) A 12,374-bp region of PMSS1 was mapped on the basis of the DNA sequence of the *cagA* genes and their flanking regions. Primer annealing sites used for PCR (filled triangles) and for DNA sequencing (empty triangles) are indicated. Three repeated *cagA* homologous areas (CHAs) were designated CHA-ud (red), CHA-u (yellow), and CHA-d (green). (D) The* cagA* copy numbers of PMSS1, G27, 26695, J99, and 7.13 were analyzed by real-time PCR using the 2^−ΔΔ*CT*^ method. *ureA* was used as a reference gene, and G27 was used as a calibrator. The bar graphs indicate the average *cagA* copy number of each strain, and error bars represent standard deviations, derived from results of 4 independent experiments.

The PMSS1 strain of *H. pylori* is capable of persistently colonizing mice and is thus a useful strain for the study of *H. pylori* infection in this animal model (52). Our attempts to construct a PMSS1 derivative containing a clean deletion of *cagA* were repeatedly unsuccessful; PCR analysis of transformants yielded unexpected banding patterns that led us to postulate that PMSS1 may contain two tandem *cagA* genes, an observation that has not been made in any other strain of *H. pylori*. To test this theory, we designed two sets of PCR primers that would allow us to detect the presence of the *cagA* gene as well as the presence of multiple adjacent *cagA* genes (Fig. 1). First, primers F and R (Fig. 1A and B, panel a) were used to confirm the presence of the *cagA* gene in the common *H. pylori* strains PMSS1, G27 (53), 26695 (54), J99 (55), and 7.13 (56) (Fig. 1B). Next, the possibility of the presence of multiple *cagA* copies was examined. PCR performed using primer dF only (Fig. 1A and B, panel b) and primer dR only (Fig. 1A and B, panel c) would detect copies of *cagA* that were arranged as inverted repeats; this PCR was negative for all strains (Fig. 1B). Finally, PCR using primers dF and dR (Fig. 1A and B, panel d) would detect copies of *cagA* that were arranged as either inverted or tandem repeats; lack of a band in PCR using only primer dF or dR would imply that the *cagA* genes were carried in tandem. The PCR with primers dF and dR generated a strong amplicon from strain PMSS1 and a weaker amplicon from strain 7.13. These data suggest that *H. pylori* strains PMSS1 and 7.13 carry multiple copies of *cagA* that are arranged as tandem repeats.

To further investigate the presence of tandem *cagA* genes in PMSS1, a 12.4-kbp region encompassing *cagA* was sequenced; the strategy was designed with the assumption that two copies of *cagA* were present. As illustrated in Fig. 1C, the sequenced region began 2,162 bp upstream of the *cagA* initiation codon and ended 1,600 bp downstream of the *cagA* termination codon; the sequence ended in the middle of the glutamate racemase gene (*glr*) open reading frame (ORF). To encompass the entire area, three separate PCR amplicons were generated and sequenced (illustrated in Fig. 1C; primers are listed in Table S1 in the supplemental material, and the sequence is available in GenBank under accession KX673184). In order to confirm the sequence of the *cagA* ORF, the ORF was amplified with primers seqF2 and seqR10 and was sequenced as illustrated in Fig. 1C. The sequence of the *cagA* ORF was identical to the sequences obtained from the three overlapping PCR amplicons. Analysis confirmed that CagA from PMSS1 contained the EPIYA-ABC motif (52), as is characteristic of Western strains of *H. pylori*. Interestingly, sequencing also revealed three *cagA* homologous areas (CHAs): a CHA located both upstream and downstream of *cagA* (CHA-ud, red, 462 bp); a CHA located only upstream of *cagA* (CHA-u, yellow, 592 bp); and a CHA located only downstream of *cagA* (CHA-d, green, 478 bp). The sequences of CHA-ud, CHA-u, and CHA-d were identical to those of the same CHAs found at other positions within the tandem repeats. However, they did not share homology with the other CHAs. In total, PMSS1 contained two copies of *cagA*, CHA-u, and CHA-d and three copies of CHA-ud (Fig. 1C). We note, however, that the sequencing strategy was not able to distinguish between more than two copies of *cagA*; interior copies would not have been amplified during the initial PCR, as the primers anneal outside the repeat region. Further, amplification using primers that anneal within the *cagA* ORF would result in indistinguishable copies of *cagA*. Thus, this PCR and sequencing method would identify identical *cagA* genes carried as direct tandem repeats. For the remainder of this article, “*cagA* repeat” is used to refer to the following DNA sequence arrangement: CHA-ud, CHA u, *cagA* ORF, and CHA-d.

As mentioned, the designed PCR and sequencing strategies were unable to determine the absolute *cagA* copy number in PMSS1. Therefore, real-time PCR was utilized to tentatively quantify the copy number of *cagA* relative to that of the urease A gene (*ureA*). The same panel of *H. pylori* strains was analyzed using chromosomal DNA as a template (Fig. 1D). The amplification efficiencies of *cagA* and *ureA* were almost equal across strains (see Fig. S1 in the supplemental material). As expected on the basis of the earlier PCR results (Fig. 1B), the relative *cagA* copy numbers in G27, 26695, and J99 were close to 1 (means ± standard deviations [SD], 1.0 ± 0.0, 1.1 ± 0.1, and 1.3 ± 0.1, respectively) but were increased in strains 7.13 and PMSS1; approximately 1.4 (± 0.1) copies of *cagA* were detected in strain 7.13, and 3.7 (± 0.1) copies were detected in strain PMSS1. These data suggest that strain 7.13 may contain a mixed population of single-copy and multiple-copy *cagA* carriers and that PMSS1 might contain more than three *cagA* copies.

10.1128/mBio.01779-16.5 Table S1  Primers used in this study. Download Table S1, XLSX file, 0.01 MB. Copyright © 2017 Jang et al. 2017 Jang et al. This content is distributed under the terms of the Creative Commons Attribution 4.0 International license.

10.1128/mBio.01779-16.1 Figure S1  Real-time PCR standard curves and *C*_*T*_ deviation of *cagA* and *ureA* in *H. pylori* strains. The standard curves were calculated by conducting real-time PCR using serial 10-fold dilutions of chromosomal DNA isolated from PMSS1, G27, 26695, J99, and 7.13; concentrations ranged from 10 ng/μl to 0.01 ng/μl. *C*_*T*_ deviations (Δ*C*_*T*_) of each strain were calculated and plotted. Download Figure S1, TIF file, 0.2 MB. Copyright © 2017 Jang et al. 2017 Jang et al. This content is distributed under the terms of the Creative Commons Attribution 4.0 International license.

### Generation of PMSS1 *H. pylori* mutant strains containing different copy numbers of *cagA.*

**FIG 2:**
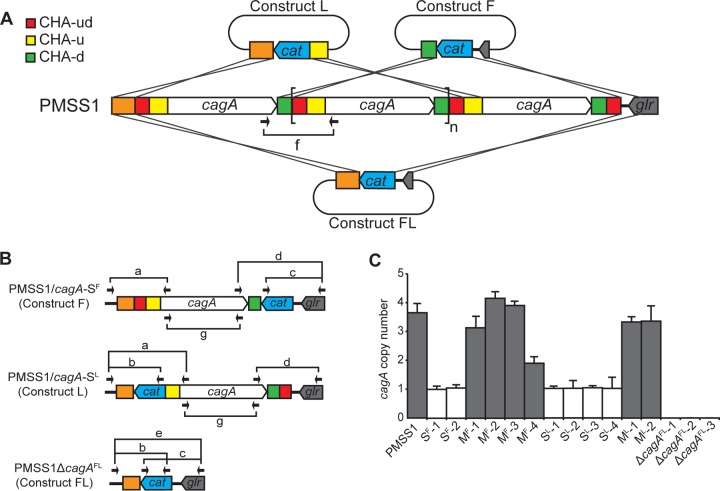
Generation and screening of PMSS1 isogenic mutant strains and determination of *cagA* copy number. (A) PMSS1 isogenic mutant strains were generated by transformation of PMSS1 with three different mutagenesis constructs: F, L, and FL. Putative homologous recombination events to generate *cagA*-S^F^, *cagA*-S^L^, and Δ*cagA*^FL^ are illustrated. The three *cagA* homologous areas (CHAs) are indicated: CHA-ud (red), CHA-u (yellow), and CHA-d (green). A single *cagA* repeat is indicated with brackets. A subscript *n* indicates that the number of repeats is undetermined. (B) A PCR-based method was used to screen for the mutant strains *cagA*-S^F^, *cagA*-M^F^, *cagA*-S^L^, *cagA*-M^L^, and Δ*cagA*^FL^. Seven different PCRs (named *a* to *g*) were used for the screen; results from PCR *f*, which identified multiple *cagA* repeats, are denoted in panel A. The alignment sites of the primers are indicated with arrows, and the primers are listed in Table S1 in the supplemental material. (C) The *cagA* copy number was determined by real-time PCR using the 2^−ΔΔ*CT*^ method. *ureA* was used as a reference gene, and S^F^-1 was used as a calibrator. The bar graphs indicate the average *cagA* copy number of each strain, and error bars represent standard deviations, derived from results of 5 independent experiments.

Increasing the copy number of a gene may serve as a mechanism to increase protein expression. To determine whether carriage of multiple copies of *cagA* results in increased CagA expression, we generated PMSS1 isogenic mutant strains that contained no, single, or multiple copies of *cagA*. Three constructs were designed to replace the first, last, or all copies of *cagA* with a chloramphenicol resistance (*cat*) cassette (Fig. 2A). Homologous recombination with construct F, which contained CHA-d and a unique 3′ region, would replace all but the first copy of *cagA* with the* cat* cassette. Strains recovered from this transformation were designated PMSS1/*cagA*-S^F^. Homologous recombination with construct L, which contained a unique 5′ region and CHA-u, would replace all but the last *cagA*. Such strains were designated PMSS1/*cagA*-S^L^. Finally, homologous recombination with construct FL, which contained a unique 5′ region and a unique 3′ region, would replace all copies of *cagA*, generating PMSS1Δ*cagA*^FL^ (Fig. 2B). Because a DNA region flanked by direct repeats can be duplicated or deleted by recombination (57), the three constructs were designed to remove any repeated CHAs flanking *cagA* or *cat*. Thus, the resulting PMSS1/*cagA*-S^F^, PMSS1/*cagA*-S^L^, and PMSS1Δ*cagA*^FL^ strains cannot undergo further recombination at this region.

Given that CHA-d and CHA-u are present at more than one location in PMSS1, the homologous recombination of constructs F and L may occur at any of the different CHA-d or CHA-u locations; the number of possible recombination sites is increased due to the carriage of multiple *cagA* repeats in PMSS1. Thus, chloramphenicol-resistant transformants may be generated by removing no *cagA* repeat or fewer *cagA* repeats than expected, depending on the site of recombination; such strains were recovered and were designated PMSS1/*cagA*-M^F^ and PMSS1/*cagA*-M^L^, on the basis of the construct used for transformation. Notably, the resulting PMSS1/*cagA*-M^F^ and PMSS1/*cagA*-M^L^ strains can still undergo further recombination due to the remaining direct repeats of CHA-u, CHA-ud, CHA-d, and the *cagA* ORF. Transformants obtained with the three constructs were screened using seven PCRs, PCR *a* to PCR *g* (Fig. 2A and B; see also Fig. S2): PCR *a* was used to identify the first 5′ *cagA*; PCR *b* to identify a *cat* gene inserted at the first 5′ *cagA* location; PCR *c* to identify the *cat* gene inserted at the last 3′ *cagA* location; PCR *d* to identify the last 3′ *cagA*; PCR *e* to identify the deletion of all *cagA* genes; PCR *f* to identify multiple copies of *cagA*; and PCR *g* to detect the presence of any copies of *cagA* and thus to confirm total deletion of *cagA*. After screening, two transformants of PMSS1/*cagA*-S^F^ (S^F^-1 and S^F^-2), four of PMSS1/*cagA*-M^F^ (M^F^-1 to M^F^-4), four of PMSS1/*cagA*-S^L^ (S^L^-1 to S^L^-4), two of PMSS1/*cagA*-M^L^ (M^L^-1 and M^L^-2), and three of PMSS1Δ*cagA*^FL^ (Δ*cagA*^FL^-1 to Δ*cagA*^FL^-3) were recovered (see Fig. S2). To confirm the results of the PCR screen, real-time PCR was performed to determine the *cagA* copy numbers (Fig. 2C). S^F^-1, S^F^-2, S^L^-1, S^L^-2, S^L^-3, and S^L^-4 contained approximately one copy of *cagA* (1.1 ± 0.1, 1.1 ± 0.1, 1.0 ± 0.1, 1.0 ± 0.2, 1.0 ± 0.1, and 1.0 ± 0.3, respectively). M^F^-1, M^F^-2, M^F^-3, and M^F^-4 contained 3.1 (± 0.3), 4.2 (± 0.2), 3.9 (± 0.1), and 1.9 (± 0.2) copies, and M^L^-1 and M^L^-2 contained 3.3 (± 0.1) and 3.4 (± 0.4) copies, respectively, indicating the presence of multiple copies of *cagA*. Strains S^F^-1, S^L^-2, M^F^-3, M^L^-1, and Δ*cagA*^FL^-2 were chosen for further characterization; the *cag* region was sequenced to confirm the expected recombination events.

10.1128/mBio.01779-16.2 Figure S2  PCR results of seven PCRs to screen mutant strains of PMSS1/*cagA*-S^F^, *cagA*-M^F^, *cagA*-S^L^, *cagA*-M^L^, and Δ*cagA*^FL^. (A) Results of PCRs *a* to *f*, which classified the strains into 5 groups: *cagA*-S^F^, *cagA*-M^F^, *cagA*-S^L^, *cagA*-M^L^, and Δ*cagA*^FL^, are shown. (B) Results of PCR *g* using primers F and R to confirm the absence of the *cagA* gene. Download Figure S2, TIF file, 1.2 MB. Copyright © 2017 Jang et al. 2017 Jang et al. This content is distributed under the terms of the Creative Commons Attribution 4.0 International license.

### More copies of *cagA* increase CagA expression and CagA phosphorylation and virulence phenotypes.

**FIG 3:**
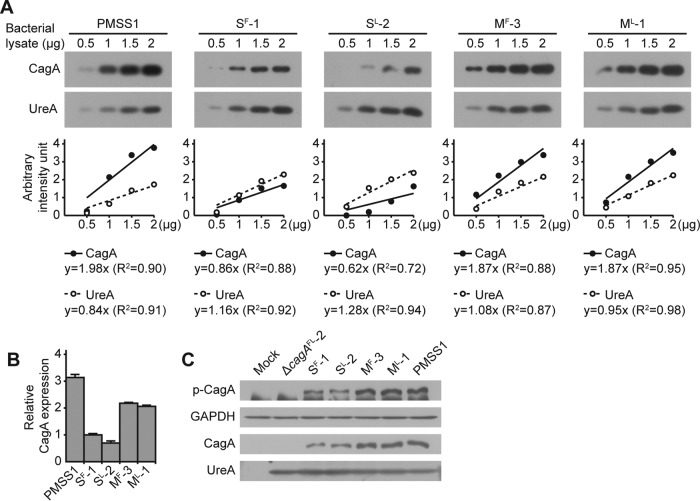
Relative levels of CagA protein and CagA phosphorylation. (A) The protein levels of CagA and UreA in lysates of *H. pylori* strains PMSS1, S^F^-1, S^L^-2, M^F^-3, and M^L^-1 were measured by Western blotting (upper panel). For each lysate, 0.5, 1, 1.5, and 2 μg of total protein were used to determine standard curves for CagA and UreA. The immunoblot images were analyzed using ImageJ software, and the values were plotted on a graph (lower panel). (B) Ratios of CagA to UreA were calculated, and each value was normalized to the value calculated for *cagA*-S^F^-1 to determine relative CagA protein levels. The bar graphs indicate average levels of CagA expression of each strain, and error bars represent standard deviations, derived from results of 2 independent experiments. (C) Lysates of AGS cells that were infected with *H. pylori* strains PMSS1, Δ*cagA*^FL^-2, S^F^-1, S^L^-2, M^F^-3, and M^L^-1 were immunoblotted for phosphorylated CagA (p-CagA), glyceraldehyde-3-phosphate dehydrogenase (GAPDH), CagA, and UreA. GAPDH and UreA were used as controls.

We hypothesized that increased numbers of copies of *cagA* would result in increased expression of CagA. Therefore, expression of CagA by PMSS1 and the S^F^-1, S^L^-2, M^F^-3, M^L^-1, and Δ*cagA*^FL^-2 transformants was measured, first within the bacterial cell and subsequently in a cell culture infection model. The levels of CagA and UreA were measured by Western blotting (Fig. 3), and strain S^F^-1 was used as a reference strain because the insertion of *cat* downstream of *cagA* resulted in a strain that carried a single copy of *cagA* (Fig. 2B). Thus, the CagA/UreA ratio of S^F^-1 was set as 1 and used for normalization of the other samples. Using this strategy, the relative expression level of CagA in strain S^L^-2 was 0.7 (± 0.1), which may indicate a slight polar effect due to the presence of the *cat* cassette upstream of *cagA*. In contrast, the relative expression levels of CagA in PMSS1, M^F^-3, and M^L^-1 were 3.1 (± 0.2), 2.2 (± 0.0), and 2.1 (± 0.1), respectively (Fig. 3A and B). These data suggest that strains of *H. pylori* that contain multiple copies of *cagA* express and produce more CagA than strains that contain only a single copy of the gene.

To measure CagA expression in a cell culture infection model, the gastric adenocarcinoma cell line AGS was infected with strains PMSS1, S^F^-1, S^L^-2, M^F^-3, M^L^-1, and Δ*cagA*^FL^-2, and the levels of total and phosphorylated CagA were measured by Western blotting (Fig. 3C). As expected, and consistent with the deletion of all copies of *cagA* from this strain, no CagA or phosphorylated CagA was detected with the Δ*cagA*^FL^-2 strain. In contrast, for strains M^F^-3, M^L^-1, and PMSS1, which each contained multiple copies of *cagA*, more phosphorylated and more total CagA was detected than was seen with strains S^F^-1 and S^L^-2. Importantly, CagA is phosphorylated only by mammalian host cell kinases; thus, more CagA is produced and successfully translocated by *H. pylori* strains carrying multiple copies of *cagA*.

**FIG 4:**
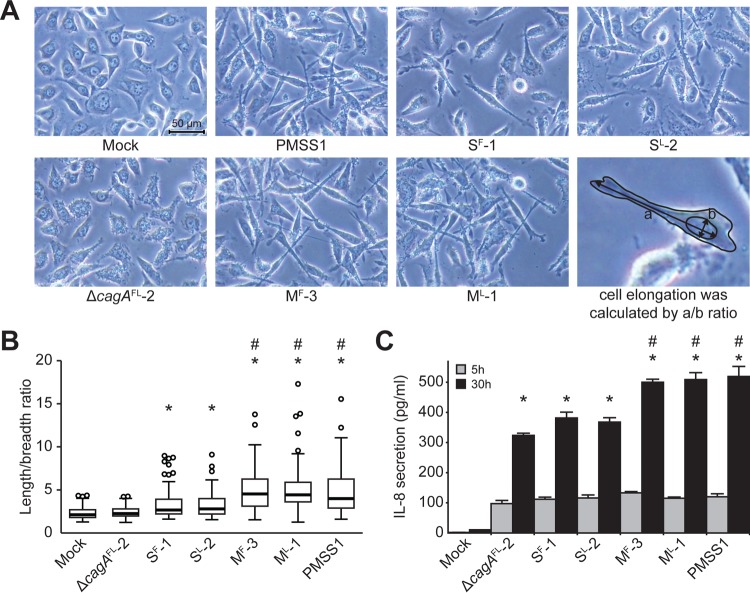
Cell elongation and IL-8 secretion in AGS cells infected by *H. pylori*. (A) AGS cells were infected with *H. pylori* strain PMSS1, Δ*cagA*^FL^-2, S^F^-1, S^L^-2, M^F^-3, or M^L^-1 at a MOI of 100 for 8 h. Micrographs were obtained under ×200 magnification. Cell elongation was calculated as the ratio of length to breadth of a cell. An example of these measurements is depicted in the figure (lower right image). (B) The cell elongation induced by each *H. pylori* strain was graphed using box plots. Thick center lines represent medians; box limits indicate the 25th and 75th percentiles; whiskers extend 1.5 times the interquartile range from the 25th and 75th percentiles; and outliers are represented by open-circle dots. *n =* 100 for each group. *, *P* < 0.05 (compared to the results of both the mock-infected and Δ*cagA*^FL^-2 groups); #, *P* < 0.05 (compared to the results obtained for both the *cagA*-S^F^-1 and *cagA*-S^L^-2 groups). (C) AGS cells were infected with *H. pylori* strain PMSS1, Δ*cagA*^FL^-2, S^F^-1, S^L^-2, M^F^-3, or M^L^-1 at a MOI of 10 for 5 h or 30 h. Secretion of IL-8 was measured at 5 h and 30 h postinfection. The bar graphs indicate average levels of IL-8 secretion of AGS cells infected with each strain, and error bars represent standard deviations, derived from results of 3 independent experiments. *, *P* < 0.05 (compared to the results from the mock-infected group); #, *P* < 0.05 (compared to the results obtained with both *cagA*-S^F^-1 and *cagA*-S^L^-2 groups).

After translocation into mammalian cells, CagA causes host cell elongation and induces expression and secretion of IL-8. To assess the effect of *cagA* copy number on these virulence phenotypes, AGS cells were again infected with strains PMSS1, S^F^-1, S^L^-2, M^F^-3, M^L^-1, and Δ*cagA*^FL^-2 (Fig. 4). Cell elongation was measured by assessing the length/breadth ratio of 100 random AGS cells on micrographs. Significantly different levels of cell elongation were found among the groups (*F*[6, 693] = 38.44, *P* < 0.001, where we report degrees of freedom as *F*[between-groups, within-groups]). Strain PMSS1 significantly induced cell elongation compared with noninfected control cells (Mock, Fig. 4A). The average length/breadth ratio (± SD) of the mock control was 2.29 (± 0.70), while the ratio calculated for PMSS1 was 5.01 (± 2.88) (Fig. 4B). Similarly to the mock infection, strain PMSS1Δ*cagA*^FL^-2 exhibited a ratio of 2.40 (± 0.67). Thus, deletion of *cagA* resulted in loss of cell elongation. S^F^-1 and S^L^-2, which each contained a single copy of *cagA*, induced cell elongation ratios of 3.39 (± 2.04) and 3.26 (± 1.45), respectively. These ratios were statistically significantly different from those determined for the mock infection and strain Δ*cagA*^FL^-2 groups. Strains PMSS1, M^F^-3, and M^L^-1, which each contained multiple copies of *cagA*, induced significantly higher levels of cell elongation (5.01 [± 2.88], 4.92 [± 2.37], and 5.09 [± 2.52], respectively) than all other strains. Thus, *H. pylori* strains containing multiple copies of *cagA* are able to induce higher levels of cell elongation.

Next, the levels of secreted IL-8 were measured in AGS cells infected with strain PMSS1, S^F^-1, S^L^-2, M^F^-3, M^L^-1, or Δ*cagA*^FL^-2 (Fig. 4C). Infections were carried out for 5 h and 30 h. Due to the presence of the T4SS, all strains, including strain Δ*cagA*^FL^-2, significantly induced IL-8 expression compared to the mock-infected control. As expected, since CagA-dependent effects on IL-8 secretion occur at later time points (34), there were no significant differences among any of the six infected groups at 5 h (*F*[6, 14] = 127.86, *P* < 0.001). In contrast, at 30 h, CagA-dependent effects were more evident (*F*[6, 14] = 266.75, *P* < 0.001) (Fig. 4C). While strains *cagA-*S^F^-1 and *cagA-*S^L^-2 induced higher levels of IL-8 than strain Δ*cagA*^FL^-2, the difference did not reach statistical significance. However, each of the strains that contained multiple copies of *cagA* (strains PMSS1, M^F^-3, and M^L^-1) induced significantly larger amounts of IL-8 than strains Δ*cagA*^FL^-2, S^F^-1, and S^L^-2. Therefore, strains of *H. pylori* containing multiple copies of *cagA* are able to induce significantly higher levels of IL-8 expression. *En masse*, these data suggest that carriage of multiple copies of *cagA* leads to increased expression and translocation of the CagA toxin, which subsequently results in more pronounced virulence phenotypes.

### Dynamic variation of *cagA* repeat numbers in PMSS1 *H. pylori.*

To begin to address the mechanism by which the PMSS1 strain came to carry multiple copies of *cagA*, we next sought to determine whether the PMSS1 population is homogeneous or heterogeneous in terms of *cagA* copy number. To this end, several hundred single colonies of PMSS1 were isolated and the *cagA* copy number for each was determined using colony PCR (Table 1; see also Fig. S3). This method was chosen to eliminate the additional culture time required for traditional genomic DNA isolation, as we anticipated that the number of *cagA* repeats could change temporally during culture. As shown in Fig. S3, five colonies that had a single *cagA* gene, as determined by real-time PCR, also generated a faint amplicon using PCR *h*; this suggests the presence of multiple *cagA* repeats (see Fig. S3). Furthermore, colonies that contained a single *cagA* gene or multiple *cagA* repeats, as determined by real-time PCR, also generated a strong amplicon using PCR *i*; this indicates the presence of *cagA*. However, these colonies also showed a faint amplicon using PCR *j*, which detected the Δ*cagA* deletion (Fig. S3). These results suggest that even during single-colony growth, the members of a minor population of *H. pylori* undergo a change in *cagA* copy number, forming a heterogeneous population. For downstream analysis of these colonies, the major population was used to assign the *cagA* gene number of the colony. Overall, among 389 first-passage colonies, 333 (85.6%) colonies carried multiple copies of *cagA*, 54 (13.8%) colonies carried a single *cagA* gene, and 2 (0.5%) colonies carried no *cagA*. Thus, the PMSS1 population was heterogeneous.

10.1128/mBio.01779-16.3 Figure S3  Genotypic analysis of multiple copies of *cagA* in single-colony derivatives of PMSS1 using colony PCR and colony real-time PCR. (A) Three PCRs, named *h* to *j*, were used for this screen; the primers are listed in Table S1. PCR *h* was used to detect multiple *cagA* repeats (putative amplicon, 1,643 bp); PCR *i* was used to detect the *cagA* gene (putative amplicon, 540 bp); and PCR *j* was used to confirm the absence of *cagA* (putative amplicon, 851 bp). (B) Representative results from the colony PCR are shown. h, detection of multiple *cagA* repeats; i, detection of the *cagA* gene; j, confirmation of the absence of the *cagA* gene. (C) Relative *cagA* copy numbers were determined by colony real-time PCR. Download Figure S3, TIF file, 0.4 MB. Copyright © 2017 Jang et al. 2017 Jang et al. This content is distributed under the terms of the Creative Commons Attribution 4.0 International license.

**TABLE 1:** Copy number of *cagA* in single colonies isolated from PMSS1, 1-107 (*cagA*-S), and 1-100 (*cagA*-M4)

Original strain	No. (%) of isolates in indicated *cagA* copy number category		
	Multiple	Single	None
PMSS1	333 (85.6)	54 (13.8)	2 (0.5)
1-107 (*cagA*-S)	1 (0.5)	190 (99.5)	0 (0.0)
1-100 (*cagA*-M4)	185 (98.4)	3 (1.6)	0 (0.0)

**FIG 5:**
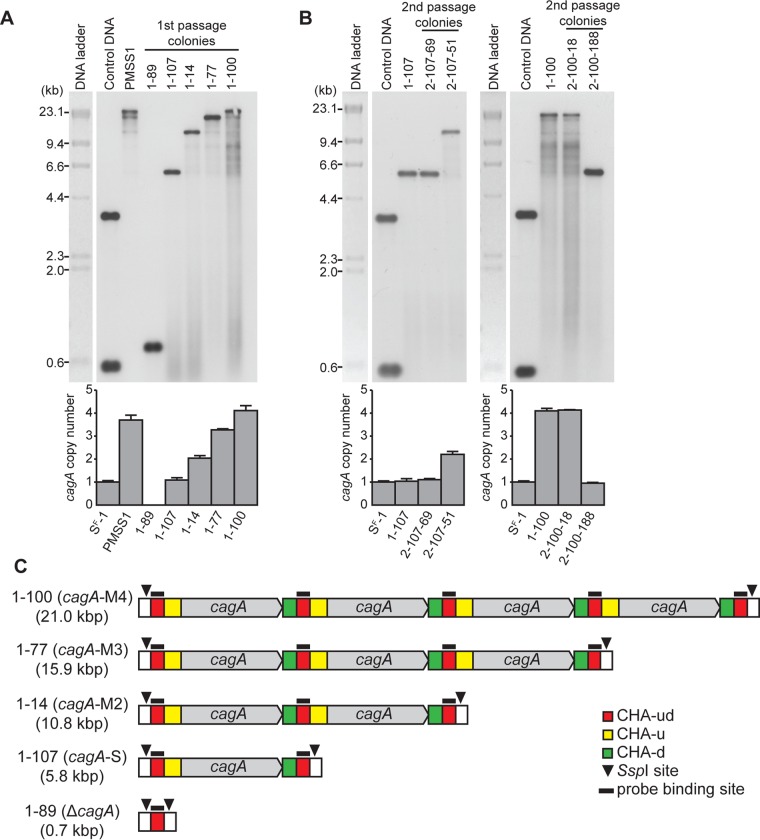
Identification of *cagA* copy number using Southern blot analysis. (A) Southern blot (upper panel) and real-time PCR (lower panel) analysis of PMSS1 and of single-colony derivatives 1-89, 1-107, 1-14, 1-77, and 1-100 at first passage from PMSS1. Control DNA used in the Southern blot was a mixture of a 3.5-kbp fragment that was linearized by SphI digestion of a pGEM clone of the hybridization probe and a 0.5-kbp fragment liberated by digestion of the same clone with EcoRI. Strain *cagA*-S^F^-1 was used for normalization of real-time PCR. The bar graphs indicate average *cagA* copy numbers, and error bars represent standard deviations, derived from results of 3 independent experiments. (B) Southern blot (upper panel) and real-time PCR (lower panel) analysis of the 1-107 and 1-100 colonies at first passage and of the single-colony derivatives 2-107-69 and 2-107-51 and single-colony derivatives 2-100-18 and 2-100-188 at second passage. (C) Schematic representation of the *cagA* repeats of PMSS1 derivatives *cagA*-M4, *cagA*-M3, *cagA*-M2, *cagA*-S, and Δ*cagA*. Three CHAs (CHA-ud, CHA-u, and CHA-d), SspI cleavage sites, and probe binding sites are indicated.

As mentioned previously (Fig. 1A), the PCR method used to type the strains was unable to differentiate between two copies of *cagA* and more copies. Thus, a subset of the colonies were also analyzed by real-time PCR and categorized into strains carrying no (strain Δ*cagA*), one (strain *cagA*-S), two (strain *cagA*-M2), three (strain *cagA*-M3), and four (strain *cagA*-M4) copies of *cagA* (data not shown). On the basis of this categorization, strains 1-89 (Δ*cagA*), 1-107 (*cagA*-S), 1-14 (*cagA*-M2), 1-77 (*cagA*-M3), and 1-100 (*cagA*-M4) were each selected for further analysis. First, the *cagA* region of each strain was sequenced. As expected, strains 1-89 (accession no. KX673186) and 1-107 (accession no. KX673185) were shown to carry no and one copy of *cagA*, respectively. The *cagA* repeats of strains 1-14, 1-77, 1-100, and PMSS1 were identical to each other and could not be distinguished by PCR and sequencing. Next, the PMSS1 strain and these other strains were used for Southern blot analysis performed with a probe that was specific for CHA-ud (Fig. 5A and C). As expected on the basis of predicted restriction patterns (Fig. 5C), bands were detected at 0.7 kbp for strain 1-89 (Δ*cagA*) and 5.8 kbp for strain 1-107 (*cagA*-S). Similarly, bands were detected at 10.8, 15.9, and 21.0 kbp for strains 1-14, 1-77, and 1-100, respectively. Of note, the PMSS1 strain showed detectable bands at 21.0 kbp, 15.9 kbp, and (faintly) 10.8 kbp, which correspond to the sizes expected for carriage of 4, 3, and 2 copies of *cagA*, respectively (indicated as *cagA*-M4, *cagA*-M3, and *cagA*-M2 in Fig. 5C). Thus, the Southern blot data further support the idea that the PMSS1 population is heterogeneous.

We next investigated whether the *cagA* copy number could dynamically change from a single copy to multiple copies and vice versa. To this end, strains 1-107 (*cagA*-S) and 1-100 (*cagA*-M4) were each passaged a second time, and the second-passage colonies were screened by PCR to detect any change in *cagA* copy number (Table 1; see also Fig. 5B). A total of 191 second-passage colonies from parental strain 1-107 (*cagA*-S) were screened. Of these colonies, one (0.5%, colony 2-107-51) changed from a single copy to multiple copies of *cagA*, while 190 (99.5%) colonies retained a single copy of *cagA*. On the other hand, of 188 second-passage colonies from parental strain 1-100 (*cagA*-M4), three colonies (1.6%) (for example, colony 2-100-188) changed to a single copy of *cagA*, while 185 colonies (98.4%) (for example, colony 2-100-18) retained multiple copies of *cagA*. Southern blot analysis revealed that strain 2-107-51 carried two copies of *cagA*, while strains 2-100-188 and 2-100-18 carried four copies and one copy of *cagA*, respectively. Thus, the *cagA* gene in PMSS1 is capable of dynamically changing copy number. We note that among the 379 second-passage colonies that we analyzed from both 1-107 (*cagA*-S) and 1-100 (*cagA*-M4) parental colonies, we did not identify any *H. pylori* isolates without any copies of the *cagA* gene. This may indicate that the recombination events required to generate this complete-loss genotype occur at a low frequency.

### Clinical isolates possess multiple copies of *cagA.*

**FIG 6:**
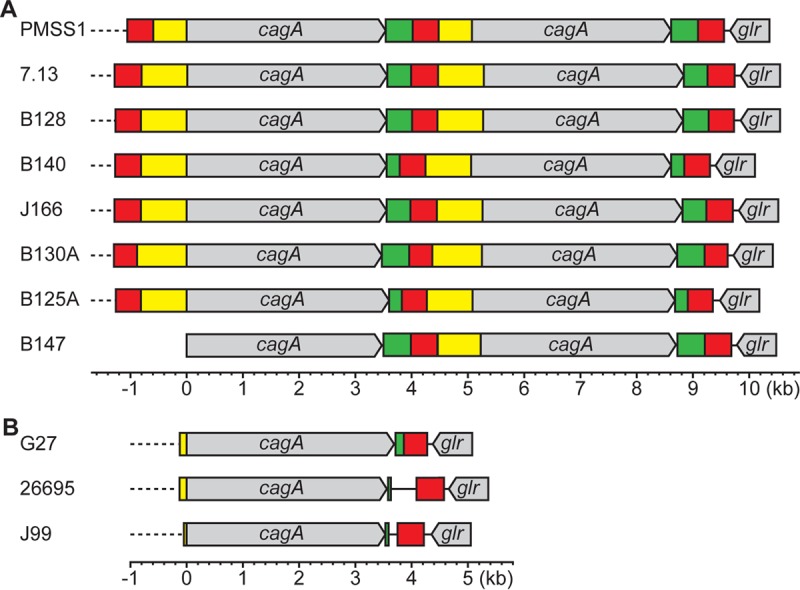
Comparison of multiple copies of *cagA* in clinical isolates. (A) Schematic representation of the *cagA* repeats in PMSS1 and 7.13 and in six clinical isolates containing multiple *cagA* repeats. In strain B147, the region upstream of *cagA* could not be sequenced. Color categorization was made according to sequence similarity to PMSS1. The three repeating *cagA* homologous areas (CHAs), CHA-ud (red), CHA-u (yellow), and CHA-d (green), are shown. (B) Schematic representation of the *cagA* repeats in G27, 26695, and J99.

To validate that the novel finding of multiple copies of *cagA* in PMSS1 was relevant to other *H. pylori* strains, we next screened a large collection of clinical isolates that originated from South Korea and the United States (23, 41, 58–60). The presence of multiple copies of *cagA* was assessed by PCR as described for Fig. 1A, and the distribution of the results is shown in Table 2 (see also Table S2). Of 234 South Korean *H. pylori* isolates previously shown to contain *cagA* (23), 219 strains were positive for *cagA* using the F and R primers. All 219 isolates carried a single copy of the gene. In contrast, of the 80 *cagA*-containing United States *H. pylori* isolates, six (7.5%) strains harbored multiple copies of *cagA*. The differences in the associations between multiple copies of *cagA* and the geographical origin of the *H. pylori* isolates were significant (Fisher exact test, *P* < 0.001). The *cagA* repeats from these six *H. pylori* strains and from the 7.13 strain, which our previous data indicated might contain multiple copies of *cagA* (Fig. 1B and D), were sequenced next (GenBank accession no. KX673187 to KX673193); the results are schematized in Fig. 6A. For comparison, the previously published genome sequences of *H. pylori* strains G27, 26695, and J99 (GenBank accession no. CP001173, AE000511, and AE001439, respectively) were used for sequence analysis and are depicted in Fig. 6B. As expected, strain 7.13 contained multiple *cagA* repeats. Additionally, the number of examples and organization of CHA-u, CHA-ud, and CHA-d in five of the six *H. pylori* clinical strains (B128, B140, J166, B130A, and B125A) and in strain 7.13 were the same as in PMSS1; moreover, CHA-u, CHA-ud, and CHA-d were highly homologous to those found in PMSS1 (see Fig. S4). The finding of conservation suggests that this is a general pattern for the presence of multiple *cagA* repeats. Unfortunately, the sequence upstream of *cagA* could not be obtained for the sixth clinical isolate, B147. This may have been due to gene rearrangement of the 5′ *cagA* region. Of note, all three CHAs were present in G27, 26695, and J99 (Fig. 6B); however, while CHA-ud was highly homologous to the CHA-ud from PMSS1, it was not found in duplicate in these strains and existed only downstream of *cagA*. In addition, CHA-u and CHA-d showed a decreased level of homology to the CHAs found in PMSS1 (see Fig. S4). Thus, our overall analysis reveals that carriage of multiple copies of *cagA* is a phenomenon that is generalizable across a subset of laboratory strains and clinical isolates of *H. pylori*.

10.1128/mBio.01779-16.6 Table S2  *cagA* EPIYA polymorphism, *vacA* s/i/m polymorphism, *homA/B* genotype, *babA/B/C* genotype, and *cagA* repeat genotype of 80 American *H. pylori* strains. Download Table S2, XLSX file, 0.02 MB. Copyright © 2017 Jang et al. 2017 Jang et al. This content is distributed under the terms of the Creative Commons Attribution 4.0 International license.

10.1128/mBio.01779-16.4 Figure S4  Comparison of CHA sequences among *cagA* repeats. The DNA sequences were aligned and compared using the AlignX program (Invitrogen). (A) The full-length CHA-u sequences from PMSS1, 7.13, B128, B140, J166, B130A, and B125A were aligned (upper panel). The same regions from G27, 26695, and J99 were aligned to CHA-u from PMSS1 (lower panel). (B) The full-length CHA-ud sequences from PMSS1, 7.13, B128, B140, J166, B130A, B125A, G27, 26695, and J99 were aligned. (C) The full-length CHA-d sequences from PMSS1, 7.13, B128, B140, J166, B130A, and B125A were aligned (upper panel). The same regions from G27, 26695, and J99 were aligned to CHA-d from PMSS1 (lower panel). Download Figure S4, TIF file, 2.8 MB. Copyright © 2017 Jang et al. 2017 Jang et al. This content is distributed under the terms of the Creative Commons Attribution 4.0 International license.

**TABLE 2:** Association between geographical origin of *H. pylori* strains and presence of multiple *cagA* repeats

Geographical origin	No. (%) of isolates in indicated *cagA* repeat category (*n =* 299)		*P* value
	Multiple	Single	
South Korea	0 (0)	219 (100)	<0.001
United States	6 (7.5)	74 (92.5)	

### Association between *cagA* copy number, genotype, and gastric disease.

The collection of *H. pylori* clinical isolates from the United States was previously characterized for several genotypic variations: *cagA* EPIYA polymorphism, *vacA* s/i/m polymorphism, *homA*/*B* genotype, and *babA*/*B*/*C* genotype (see Table S2 in the supplemental material) (59). Given our data that suggested that increased *cagA* copy numbers lead to increased pathogenesis (Fig. 4), we next assessed whether there were any epidemiological associations between the multiple *cagA* copies and the available strain and patient data (Table 3; see also Table S3). Despite the fact that all six isolates harboring multiple copies of *cagA* were from white patients, there was no significant association between copies of *cagA* and the patient’s ethnic group. However, there was a significant association of multiple *cagA* repeats with occupancy of *bab* locus C; this was true both when the locus was measured as simply occupied or empty and when it was measured as occupied by *babA*, *babB*, or *babC* or was empty. Of the six multicopy *cagA* isolates, three (50%) strains carried a *bab* paralog at locus C. In comparison, locus C was occupied in only 5 (7%) of the 74 single-copy isolates. This may suggest that there is a functional association between the presence of a *bab* paralog at locus C and the presence of multiple *cagA* repeats.

**TABLE 3:** Significant associations of multiple *cagA* copies with genotypic variations and disease state

Parameter compared with *cagA* repeat genotype^a^ (*n =* 80)	*P* value
EPIYA-AB or EPIYA-ABC or EPIYA-ABCC or other	0.013
Empty or *babA* or *babB* or *babC* at *bab* locus C	0.005
Empty or occupied at *bab* locus C	0.012
Cancer or Barrett’s esophagus or gastric ulcer or duodenal ulcer or gastritis or esophagitis	0.036
Cancer, Barrett’s esophagus, and gastric ulcer or duodenal ulcer and gastritis or esophagitis	0.024
Cancer and gastric ulcer or duodenal ulcer and gastritis or Barrett’s esophagus and esophagitis	0.016

a The *cagA* repeat genotype was categorized as single or multiple *cagA* repeats.

10.1128/mBio.01779-16.7 Table S3  Associations of multiple *cagA* copies with ethnic groups, genotypic variations, and disease state. Download Table S3, XLSX file, 0.01 MB. Copyright © 2017 Jang et al. 2017 Jang et al. This content is distributed under the terms of the Creative Commons Attribution 4.0 International license.

Particular *cagA* polymorphisms are known to be associated with more severe disease outcomes (23, 60, 61). Furthermore, we found a significant association between multiple copies of *cagA* and EPIYA motif polymorphism (Table 3). Among the six isolates that contained multiple copies of *cagA*, two isolates carried EPIYA-AB and four isolates carried EPIYA-ABC. Notably, while two of the three isolates containing EPIYA-AB had multiple copies of *cagA*, none of the 27 strains carrying EPIYA-ABCC carried more than one copy of *cagA*.

Finally, the association between multiple copies of *cagA* and disease state was assessed (Table 3). The six isolates containing multiple *cagA* repeats were from patients diagnosed with gastric ulcer (*n =* 3), duodenal ulcer (*n =* 1), and esophagitis (*n =* 2). Despite the small number of strains carrying multiple copies of *cagA*, the distribution of single or multiple copies analyzed on the basis of individual disease state was significant (Table 3). Significant associations were also seen when various disease states were grouped on the basis of combinations of diseases that reflect differences in severity and/or anatomical location (Table 3). Three of the 6 multiple-copy *cagA* strains were from patients with gastric ulcers, of which there were only 10 in total within the entire collection. Furthermore, the fact that no strains harboring multiple copies of *cagA* were found among the large number of gastritis patients likely influences the observed statistical associations. *En masse*, these data suggest that the presence of multiple copies of *cagA* may impact the development of gastric disease.

## DISCUSSION

Despite the presence of 84 completely sequenced and assembled *H. pylori* genomes in the NCBI database, no strains of *H. pylori* have been shown to carry identical copies of *cagA*, which encodes what is arguably the most-studied virulence factor of this pathogen. However, here we describe the novel finding that some strains of *H. pylori* harbor multiple tandem copies of *cagA*. Among the strains that carry multiple *cagA* copies, PMSS1 was originally isolated from a patient with a duodenal ulcer and has recently been widely studied since it can persistently colonize mice; this provides an invaluable tool to study gastric disease development in an animal model (52). Our data indicate that the PMSS1 strain represents a heterogeneous population where individual bacterial cells carry between zero and four copies of *cagA* (Fig. 5A). At the population level, real-time PCR analysis indicated that PMSS1 contained an average of 3.7 copies of *cagA* (Fig. 1D). Characterization of individual isolates that were engineered to contain various numbers of *cagA* repeats (PMSS1/*cagA*-S^F^ and PMSS1/*cagA*-S^L^) showed that both the 5′ and 3′ *cagA* genes functionally expressed CagA (Fig. 3A). Furthermore, DNA sequence analysis showed that in strains containing multiple copies of *cagA*, these genes existed as identical tandem direct repeats that also contained three CHA types (CHA-u, CHA-ud, and CHA-d) (Fig. 1C). Remarkably, the *cagA* repeats in PMSS1 were able to expand and contract during culture in rich media (Fig. 5B); thus, even *in vitro*, *cagA* copy number can undergo dynamic change.

The existence of multiple copies of *cagA* led us to hypothesize that these strains would express more CagA, deliver more CagA to host cells, and, therefore, show increased levels of CagA-dependent virulence. Indeed, when the amount of CagA produced by PMSS1/*cagA*-S^F^-1, which contained a single copy of *cagA*, was set to 1, the relative CagA expression levels of strains harboring increasing copies of *cagA* were proportional, though not one to one. Specifically, the level of CagA expressed by strain PMSS1, with 3.7 copies of *cagA*, was 3.1; that expressed by strain PMSS1/*cagA*-M^F^-3, with 3.9 copies, was 2.2; and that expressed by strain PMSS1/*cagA*-M^L^-1, with 3.3 copies, was 2.1 (Fig. 3A). Furthermore, the *cagA* copy number was proportional to the levels of the CagA-dependent virulence phenotypes that were induced: cell elongation and late induction of IL-8. In sum, increased copies of *cagA* led to more CagA expression and, subsequently, to increased virulence.

Though the exact mechanism for expansion and contraction of the *cagA* repeats is not yet clear, an analysis of the DNA sequences of strains PMSS1Δ*cagA* and PMSS1/*cagA*-S allows us to hypothesize a potential mechanism by which this could occur. Previous studies showed that when a gene is flanked by direct repeats, the copy number of the gene can increase or decrease due to recombination between the repeats (57, 62–65). Therefore, it is likely that CHA-ud plays a critical and unique role in the generation of multiple *cagA* repeats from a homogeneous population containing only a single *cagA* repeat. This assertion is based on the fact that only CHA-ud exists as two copies in strain PMSS1/*cagA*-S; this type of repetition is necessary for standard homologous recombination to duplicate a gene. Additionally, CHA-ud is the only CHA that remained in the PMSS1Δ*cagA* strain. It is worth noting that a change from the PMSS1Δ*cagA* population to a PMSS1/*cagA*-S or PMSS1/*cagA*-M population could not happen if the PMSS1Δ*cagA* population were homogeneous. Given that CHA-ud is highly conserved among *H. pylori* strains (see Fig. S4) and that coinfection with multiple strains of *H. pylori* occurs clinically (66–69), this raises the intriguing issue of whether interstrain recombination at the *cagA* region occurs; this issue remains to be addressed.

Moreover, expansion to multiple repeats was not detected in the PMSS1/*cagA*-S^F^-1 and -S^L^-2 mutant strains. This is likely because generation of these strains left only a single copy each of CHA-ud, CHA-u, *cagA*, and CHA-d. We do note that, despite our hypothesis that CHA-ud is the central player in *cagA* change, in *H. pylori cagA*-M, all of the CHAs (CHA-u, CHA-d, and CHA-ud) and *cagA* are present at least in duplicate; thus, any of these could formally be involved in duplication or deletion of the *cagA* repeats. The frequency of recombination between repeats is proportional to the number (70, 71) and length (72) of the repeats. Our data indicated that approximately 1.6% of the PMSS1/*cagA*-M4 population recombined into the PMSS1/*cagA*-S population during one passage. However, only 0.5% of the PMSS1/*cagA*-S population recombined to form the PMSS1/*cagA*-M population, suggesting that this event is rarer. In PMSS1/*cagA*-S, only CHA-ud is present as a tandem repeat in a manner that could facilitate the recombination process. In contrast, PMSS1/*cagA*-M4 possesses four copies of *cagA*, four of CHA-u, four of CHA-d, and five of CHA-ud, all of which could possibly be involved in the recombination event. If this were the case, this would explain why the recombination rate in the PMSS1/*cagA*-M4 population was higher than that that seen in the PMSS1/*cagA*-S population. It is worth noting that the recombination frequency was measured in rich media and thus that the frequency may be different in *in vitro* cell culture or under *in vivo* conditions. Clearly, the exact mechanism of change for *cagA* copy numbers remains to be elucidated.

We analyzed the 84 complete *H. pylori* genomes but were not able to identify any strains that were shown to carry tandem repeats of *cagA*. We did note that two genome sequences, Shi470 and v225d (GenBank accession numbers CP001072 and CP001582, respectively), did show carriage of two *cagA* genes at two distant loci. However, the CagA copies in Shi470 shared only 85% homology and one of the *cagA* sequences in v225d was predicted to be a truncated pseudogene (73). Thus, our work is the first to suggest the presence of multiple functional copies of CagA in *H. pylori*. We do note that since we were able to readily identify numerous strains containing multiple repeats of *cagA*, it is highly likely that their occurrence had been previously missed in standard genome sequencing projects; this is due to the average length obtained by most high-throughput sequencing technologies combined with assembly programs that are designed to obtain a single “best fit” contig. This issue could be overcome either by technological advancements that allow greater read lengths or by significantly increasing the sequencing depth of the genome. The latter option would then require analysis of the data to identify areas showing increased sequencing coverage, which could indicate gene duplication. Indeed, this sequencing based strategy has concurrently been used to identify multiple copies of *cagA* in recently sequenced *H. pylori* strains (86). It will be of interest to see if new genome sequences generated by future DNA sequencing applications will be able to identify strains carrying multiple *cagA* repeats.

Among the relatively small number (*n* = 80) of United States clinical isolates that we analyzed, 7.5% were shown to contain multiple copies of *cagA*. In contrast, none of the 219 South Korean *H. pylori* isolates that we analyzed carried more than one copy of *cagA*. Numerous previous studies have shown that there are distinct genetic differences between *H. pylori* strains isolated in Western countries, including the United States, and those isolated in East Asian countries, including South Korea (23, 41, 58–60, 74, 75). Thus, this difference in *cagA* copy numbers between the South Korean and United States *H. pylori* isolates may not be surprising. The strains containing multiple* cagA* copies within this collection possessed various virulence factor polymorphisms and genotypes: *cagA* EPIYA polymorphism, *vacA* s/i/m polymorphism, and *homA*/*B* and *bab* genotypes (see Table S2). This indicates that these strains are not closely related to each other. Thus, to identify the origin of the multiple* cagA* copies, further future genomic analyses will be necessary. Perhaps harboring multiple copies of *cagA* may be a strategy by which *H. pylori* adapts to more diverse hosts within the population. Within this realm, it is worth mentioning that the two laboratory strains of *H. pylori* (PMSS1 and 7.13) that we showed to carry multiple copies of *cagA* are both animal-colonizing strains (52, 56). Thus, the ability to change the number of *cagA* copies may also be important for adaptation to new host environments. Clearly, the ability of *H. pylori* strains to affect genetic diversity plays a critical role in this adaptation process.

Since the *cagA* EPIYA polymorphism, *vacA* s/i/m polymorphism, *homA*/*B* genotype, and *babA*/*B*/*C* genotype of the 80 *H. pylori* clinical isolates from the United States were previously identified (see Table S2) (59), we were able to easily investigate whether the presence of multiple copies of *cagA* had any association with other virulence factors. To this end, we found a positive association with occupancy of *bab* locus C. Interestingly, Hennig et al. (48) previously reported an association of *babA* carried at any *bab* locus with the presence of *cagA* in United States* H. pylori* isolates. Furthermore, Kim et al. (59) described an association between the genotype of the presence of the *bab* gene at locus A and the *cagA* EPIYA-ABD genotype in *H. pylori* isolates from Korean and American populations. *En masse*, these studies suggest that there is a close relationship between the *bab* and *cagA* genotypes. The molecular basis for this relationship remains to be elucidated.

The other genotype which was shown to be associated with the presence of multiple *cagA* copies was the CagA EPIYA type. Multiple copies of *cagA* appeared only in strains carrying no or single EPIYA-C motifs. Since CagA variants containing an increased number of EPIYA-C motifs are known to correlate with more virulent disease characteristics (15, 24, 25), carriage of multiple copies of *cagA* in strains containing the AB or ABC motifs might provide a mechanism to compensate for the decreased virulence abilities of those CagA variants. Thus, the dynamic expansion and contraction of *cagA* copy number may serve as a novel mechanism by which *H. pylori* modulates gastric disease development.

## MATERIALS AND METHODS

### Bacterial strains and cultures.

*H. pylori* strains PMSS1, G27, 26695, J99, and 7.13 and the 15 PMSS1 isogenic mutant strains containing different numbers of *cagA* repeats were cultured and stored as previously described (76). Briefly, all *H. pylori* strains were grown on horse blood agar plates supplemented with antibiotics and stored at −80°C until use. Chloramphenicol was added to the horse blood agar plates or liquid culture medium at a concentration of 8 μg/ml for cultures of PMSS1 derivatives that contained the *cat* cassette. For infection of mammalian cells to measure cell elongation and IL-8 induction and for immunoblot assays, *H. pylori* strains were prepared as previously described (23), with minor modifications. *H. pylori* strains were initially cultured in brucella broth (BD, Franklin Lakes, NJ) containing 10% fetal bovine serum (FBS) (Gibco, Grand Island, NY, USA) and 10 μg/ml vancomycin (Duchefa, Haarlem, Netherlands) for 24 h and were then inoculated into new media to obtain an optical density of 0.05 at 600 nm. These cultures were grown for 18 h with shaking at 110 rpm. All *H. pylori* strains were cultured at 37°C under microaerophilic conditions generated by an Anaeropack-Microaero gas-generating system (Mitsubishi Gas Chemical, Tokyo, Japan).

### AGS cell culture.

AGS (ATCC CRL-1739), a human gastric adenocarcinoma epithelial cell line, was maintained in RPMI 1640 (Gibco) media supplemented with 10% FBS, 100 U/ml penicillin, and 100 µg/ml streptomycin (Gibco). Cells were cultured at 37°C in a water-saturated 5% CO_2_ air atmosphere.

### Clinical *H. pylori* isolates.

A total of 314 clinical *H. pylori* isolates obtained from South Korea and the United States were used in this study. The 234 South Korean *H. pylori* clinical isolates were a subset of a collection of South Korean strains used in previous studies (23, 41, 58, 60), and the 80 isolates from the United States were the population described in a previous study (59).

### *cagA* repeat genotyping.

A PCR-based method was designed to identify the presence of multiple *cagA* repeats and the orientation of multiple *cagA* genes in *H. pylori* (Fig. 1A). The primers used for the genotyping of the *cagA* repeats are listed in Table S1 in the supplemental material. Chromosomal DNA was extracted from *H. pylori* strains as previously described (77) and was used as a template for PCR. Amplification with primer set F and R was used to determine the presence of the *cagA* gene (Fig. 1A, panel a). Primers F and R were designed to match a conserved *cagA* region in *H. pylori* strains PMSS1, G27, 26695, J99, and 7.13 on the basis of genome sequences available in GenBank. The dF and dR primers are complementary to the R and F primers, respectively. Amplification with just the dF or dR primer was used to determine tail-to-tail or head-to-head orientation of multiple *cagA* inverted repeats (Fig. 1A, panels b and c), respectively. Amplification with the primer set of dF and dR was used to identify adjacent multiple *cagA* repeats (Fig. 1A, panel d).

### Real-time PCR.

Real-time PCR was conducted to determine numbers of *cagA* copies in the *H. pylori* strains. *ureA* was used as a reference gene to quantify the relative number of repeats of *cagA*. Specific primers for *cagA* (RTcagAF and RTcagAR) and *ureA* (RTureAF and RTureAR) were designed to yield 145-bp and 142-bp amplicons, respectively. Primers used for the real-time PCR are listed in Table S1. The relative numbers of *cagA* copies were quantified using the 2^−ΔΔ*CT*^ method (78, 79), where −ΔΔ*C*_*T*_ = Δ*C*_*T*_ of target − Δ*C*_*T*_ of calibrator; Δ*C*_*T*_ = *C*_*T*_ of *cagA* − *C*_*T*_ of *ureA*; and *C*_*T*_ = threshold cycle. G27 was used as the calibrator for *cagA* copy number determinations for the other wild-type strains, and strain *cagA*-S^F^-1 was used as the calibrator for the copy number determinations for PMSS1-derived mutant strains and single-colony isolates. The real-time PCR analysis was performed using SYBR Premix *Ex Taq* (TaKaRa, Kusatsu, Japan) on a 7300 real-time PCR system (Applied Biosystems, Foster City, CA) according to the instructions of the manufacturers. Briefly, samples were initially denatured for 30 s at 95°C and then amplified for 40 cycles of 5 s at 95°C and 31 s at 60°C. The data for the fluorescence signal were collected at the end of the 60°C step in each cycle. Melting curves of amplicons from *cagA* and *ureA* were analyzed to exclude amplification of nonspecific products. The amplification efficiencies of *cagA* and *ureA* for each strain were determined with a standard curve. The standard curve was generated by applying a 10-fold serial dilution of chromosomal DNA to real-time PCR quantification. The data were analyzed using 7300 System SDS software version 1.4 (Applied Biosystems) and are presented as means ± SD of the results from the indicated replicate.

### Generation of PMSS1 *cagA* isogenic mutant strains.

Three constructs targeting the PMSS1 *cagA* gene at different positions were generated (Fig. 2). Construct F, which replaces the *cagA* gene at the last 3′ position with a *cat* cassette, was made as follows. Amplicons generated from PCRs using a primer set of cagAF-5′F and cagAF-5′RXS and a primer set of cagAF-3′FXS and cagAdownR were fused by splicing by overlap extension (SOE) PCR (80, 81). The fused amplicon contained XhoI and SmaI sites in the overlap-joining region. The fused amplicon was inserted into a pGEM-T Easy vector system (Promega, Madison, WI) by TA cloning. A *cat* cassette, liberated from plasmid pKJMSH (82) by double digestion with XhoI and SmaI, was ligated with the XhoI-SmaI doubly digested plasmid that contained the fused amplicon. The other two constructs were made with the same process except they used different primers for the SOE PCR. Construct L, which replaces the *cagA* gene at the first 5′ position with a *cat* cassette, was made using the primer set of cagAupF and cagAL-5′RXS and the primer set of cagAL-3′FXS and cagAL-3′R. Construct FL, which replaces all *cagA* genes with a *cat* cassette, was made using the primer set of cagAupF and cagAL-5′RXS and the primer set of cagAF-3′FXS and cagAdownR. The three constructs were then introduced into PMSS1 by natural transformation (83). Transformants were selected on horse blood agar plates supplemented with 8 µg/ml chloramphenicol, and the double homologous recombination of the construct into single-colony isolates of chloramphenicol-resistant *H. pylori* was verified by seven PCRs (Fig. 2A and B; see also Fig. S2 in the supplemental material) and DNA sequencing. The primers used to generate and verify the constructs are listed in Table S1. PCR *a* with primers seqF1 and dR was used to identify the first 5′ *cagA*, PCR *b* with primers seqF1 and catR was used to identify the *cat* gene inserted at the first 5′ *cagA* location, PCR *c* with primers catF and seqR12 was used to identify the *cat* gene inserted at the last 3′ *cagA* location, PCR *d* with primers dF and seqR12 was used to identify the last 3′ *cagA*, PCR *e* with primers seqF1 and seqR12 was used to identify the deletion of all *cagA* genes, and PCR *f* with primers dF and dR was used to identify multiple *cagA* repeats. Finally, PCR *g* with primers F and R was used to identify the presence of the *cagA* gene. The number of *cagA* genes in each colony was determined by real-time PCR.

### Cell elongation assay.

AGS cells were seeded onto 6-well cell culture plates at a density of 4 × 10^5^ cells per well and were then incubated for 1 day at 37°C. At 2 h prior to infection, cells were washed with phosphate-buffered saline (PBS) and the medium was changed to 2 ml of RPMI 1640 containing 2% FBS and no antibiotics. Liquid cultures of *H. pylori* were resuspended in RPMI 1640 containing 2% FBS, and AGS cells were infected at a multiplicity of infection (MOI) of 100. At 8 h postinfection, cells were fixed with 4% paraformaldehyde. Images of the cells were taken using a CKX41 inverted microscope and a DP20 microscope camera (Olympus, Tokyo, Japan) under ×200 magnification. One hundred cells were randomly selected from each well, and cell elongation was calculated by dividing the length of the longest protrusion of a cell by the breadth of the cell, as previously described (84). The length of the longest protrusion was defined as the length of the line that connects the end of the protrusion and the farthest border of the nucleus of a cell, and the breadth was defined as the diameter of the nucleus perpendicular to the line used to measure the length of the cell (Fig. 4A). The length and the breadth were measured using ImageJ software version 1.47 (National Institutes of Health, Bethesda, MD), and then averaged ratios in each group were statistically analyzed. Values corresponding to the elongation of cells induced by each *H. pylori* mutant strain are presented as box plots generated using the BoxPlotR website (http://boxplot.tyerslab.com), which utilizes R software to analyze data and generate plots.

### IL-8 secretion assay.

AGS cells were seeded onto 6-well cell culture plates at a density of 4 × 10^5^ cells per well and were then incubated for 2 days at 37°C. At 2 h prior to infection, cells were washed with PBS and the medium was changed to 2 ml of RPMI 1640 containing 2% FBS and no antibiotics. Liquid cultures of *H. pylori* were resuspended in RPMI 1640 containing 2% FBS, and AGS cells were infected at a MOI of 10; the lower MOI, compared to that used for the cell elongation assays, was used to prevent significant levels of cell death during the longer incubation required in the IL-8 assay. The infection was allowed to proceed for 5 h as a means to measure *cag* PAI-dependent immediate IL-8 secretion and for 30 h as a means to measure CagA-dependent late IL-8 secretion (34). At 5 and 30 h postinfection, 1 ml of cell culture medium was taken and centrifuged at 12,000 × *g* for 10 min at 4°C, and the supernatant was used for IL-8 enzyme-linked immunosorbent assay (ELISA). Assays were performed using human IL-8 ELISA Max Deluxe (BioLegend, San Diego, CA) following the manufacturer’s instructions, and absorbance was measured using an Epoch microplate spectrophotometer (BioTek, Winooski, VT). Data were presented as means ± SD of results from 3 independent replicates.

### Immunoblot assay.

To prepare *H. pylori* bacterial lysates, 1.5 ml of overnight liquid culture of the various *H. pylori* strains was pelleted and then lysed with 100 µl of cell lysis buffer (Cell Signaling, Inc., Danvers, MA) supplemented with protease inhibitor cocktail (Roche, Basel, Switzerland). To prepare lysates of infected cells, AGS cells were seeded onto 6-well cell culture plates at a density of 4 × 10^5^ cells per well and were then incubated for 2 days at 37°C. At 2 h prior to infection, cells were washed with PBS and the medium was changed to 2 ml of RPMI 1640 containing 2% FBS and no antibiotics. Liquid cultures of *H. pylori* were resuspended in RPMI 1640 containing 2% FBS, and AGS cells were infected at a MOI of 100. At 5 h postinfection, cells were washed with PBS and then lysed with 100 µl of cell lysis buffer supplemented with protease inhibitor cocktail. Protein concentrations of bacterial lysates and infected cell lysates were measured using Pierce bicinchoninic acid (BCA) protein assay reagent (Thermo Fisher Scientific, Waltham, MA). The indicated amount of each sample was separated by sodium dodecyl sulfate-polyacrylamide gel electrophoresis and then transferred to a polyvinylidene fluoride membrane (Merck Millipore, Darmstadt, Germany). To detect CagA, phosphorylated CagA, UreA, and GAPDH (glyceraldehyde-3-phosphate dehydrogenase), membranes were probed by the use of rabbit polyclonal anti-CagA antibody b-300 (Santa Cruz Biotechnology, Dallas, TX), mouse monoclonal anti-phosphotyrosine antibody pY99 (Santa Cruz Biotechnology), rabbit polyclonal anti-UreA antibody b-234 (Santa Cruz Biotechnology), and rabbit polyclonal anti-GAPDH antibody (Koma Biotech, Seoul, South Korea), respectively. These membranes were further probed with goat anti-mouse IgG-horseradish peroxidase (IgG-HRP) (Santa Cruz Biotechnology) or goat anti-rabbit IgG-HRP (Santa Cruz Biotechnology). Antibodies against CagA, phosphorylated CagA, UreA, and GAPDH were diluted to 1:10,000 in 3% bovine serum albumin dissolved in Tris-buffered saline with 0.1% Tween 20 (TBST), and HRP-conjugated antibodies were diluted to 1:10,000 in 3% skim milk dissolved in TBST. Probed membranes were then developed using WesternBright enhanced chemiluminescence-HRP (ECL-HRP) substrate (Advansta, Menlo Park, CA, USA) on X-ray film (Agfa, Mortsel, Belgium). Relative expression levels of CagA were measured as previously described (85). Briefly, developed films were scanned to image files, intensities of blots on the images were measured using ImageJ software version 1.47, and the values were plotted on a graph. A ratio of CagA to UreA was calculated, and each value was normalized to the value of *cagA*-S^F^-1 to determine relative CagA protein levels.

### DNA sequencing.

Sanger dideoxy DNA sequencing was performed at Cosmo Genetech Co., Ltd. (Seoul, South Korea). The DNA sequencing primers are listed in Table S1. The resulting DNA sequences were analyzed using Vector NTI version 9.1 (Invitrogen, Carlsbad, CA) and Sequencher 5.1 (Gene Codes, Ann Arbor, MI).

### Genotyping of *H. pylori* single colonies by colony PCR.

A culture of PMSS1 and its derivatives was diluted in brucella broth and was plated onto horse blood agar plates. Plates were incubated for 4 days under microaerobic conditions until single colonies appeared, which was considered to represent one passage. Single colonies were picked up and streaked onto a new blood agar plate for further culture. The cultures were used for isolation of genomic DNA and frozen stocks.

Colonies were transferred into 20 μl of Tris-EDTA (TE) buffer (pH 8.0) and then were heated at 99°C for 3 min. The supernatant of the TE buffer after centrifugation was used as the template for both PCR and real-time PCR to analyze *cagA* copy numbers in single colonies of PMSS1 and its derivatives.

A colony PCR was designed to identify the absence of or the presence of single or multiple *cagA* genes in colonies. Primers specific to PMSS1 were designed for more efficient and more accurate PCR (Table S1). The primer alignment sites are indicated in Fig. S3A. Three sets of colony PCRs were performed in parallel: PCR *h* with primers dF2 and dR2 was used to detect the presence of multiple *cagA* genes; PCR *i* with primers F2 and R2 was used to detect the presence or absence of *cagA*; and PCR *j* with primers cagAupF2 and cagAdownR2 was used to confirm the absence of *cagA*. PCR *h* was carried out as follows: a cycle at 94°C for 3 min; 35 cycles of 94°C for 15 s, 54°C for 15 s, and 72°C for 90 s; a final elongation step at 72°C for 10 min. For PCRs *i* and *j*, the same PCR parameters were used except a 60-s extension time was used instead of 90 s; this change decreased amplification of the larger (>5.5-kbp) PCR product generated from genomes containing *cagA* with PCR *j*. Thus, PCRs *i* and *j* generated a 540-bp amplicon to detect *cagA* and an 851-bp amplicon from a *cagA* deletion, respectively. A colony real-time PCR was performed to determine *cagA* copy numbers in the single colonies. The colony-boiled TE buffer supernatant was used as a template. The colony real-time PCR was conducted as described for the real-time PCR.

### Southern blot assay.

A total of 0.5 μg of chromosomal DNA from each sample was digested with restriction enzyme SspI (Thermo Fisher Scientific) and resolved on a 0.8% agarose gel for 12 h at 25 V. SspI was used because the SspI site is not found within the *cagA* repeat region (Fig. 5C). The DNA fragments were transferred from the gel to a positively charged nylon membrane (Roche) via downward capillary action using alkaline transfer. A probe was generated using a PCR digoxigenin (DIG) Probe synthesis kit (Roche) according to the manufacturer’s instructions. Briefly, chromosomal DNA of PMSS1 was used as a template to amplify CHA-ud by PCR using the primer set of probe-F and probe-R (Table S1) and the 462-bp amplicon was cloned into a pGEM-T Easy vector system, resulting in the pGEM probe. The pGEM probe containing the amplicon was purified and used as a DNA template to synthesize the DIG-labeled probe. The probe was designed to target the CHA-ud sequence (Fig. 5C). Prehybridization and hybridization were performed at 45°C for 30 min and overnight in DIG Easy Hyb solution (Roche), respectively. Washing and blocking steps were done using a Wash buffer set and a Block buffer set (Roche), respectively, and the detection step was accomplished using anti-digoxigenin-alkaline phosphatase (AP)/Fab fragments and CSPD chemiluminescence substrate (Roche).

### Statistical analysis.

Statistical analyses were performed using the IBM SPSS statistics 23 program (IBM, Armonk, NY). The results of the cell elongation assay and IL-8 induction assay were analyzed by one-way analysis of variance followed by Tukey’s *post hoc* test. *P* values were presented with *F* values and degrees of freedom (for between-group and within-group comparisons). The Fisher exact test was used to analyze the association between the geographical origin of *H. pylori* clinical isolates and the presence of multiple *cagA* repeats and between *cagA* copy number, genotype, and gastric disease. A *P* value of less than 0.05 was considered to be statistically significant.

### Accession number(s).

The sequences for the *cagA* gene(s) and their flanking regions from 10 strains have been deposited in GenBank under accession no. KX673184 to KX673193.
