# Supplementary figures and images for: Dynamic Expansion and Contraction of cagA Copy Number in Helicobacter pylori Impact Development of Gastric Disease
Source: mBio. 2017 Feb 21;8(1):e01779-16. doi: 10.1128/mBio.01779-16 (PMC5358911; doi:10.1128/mBio.01779-16)

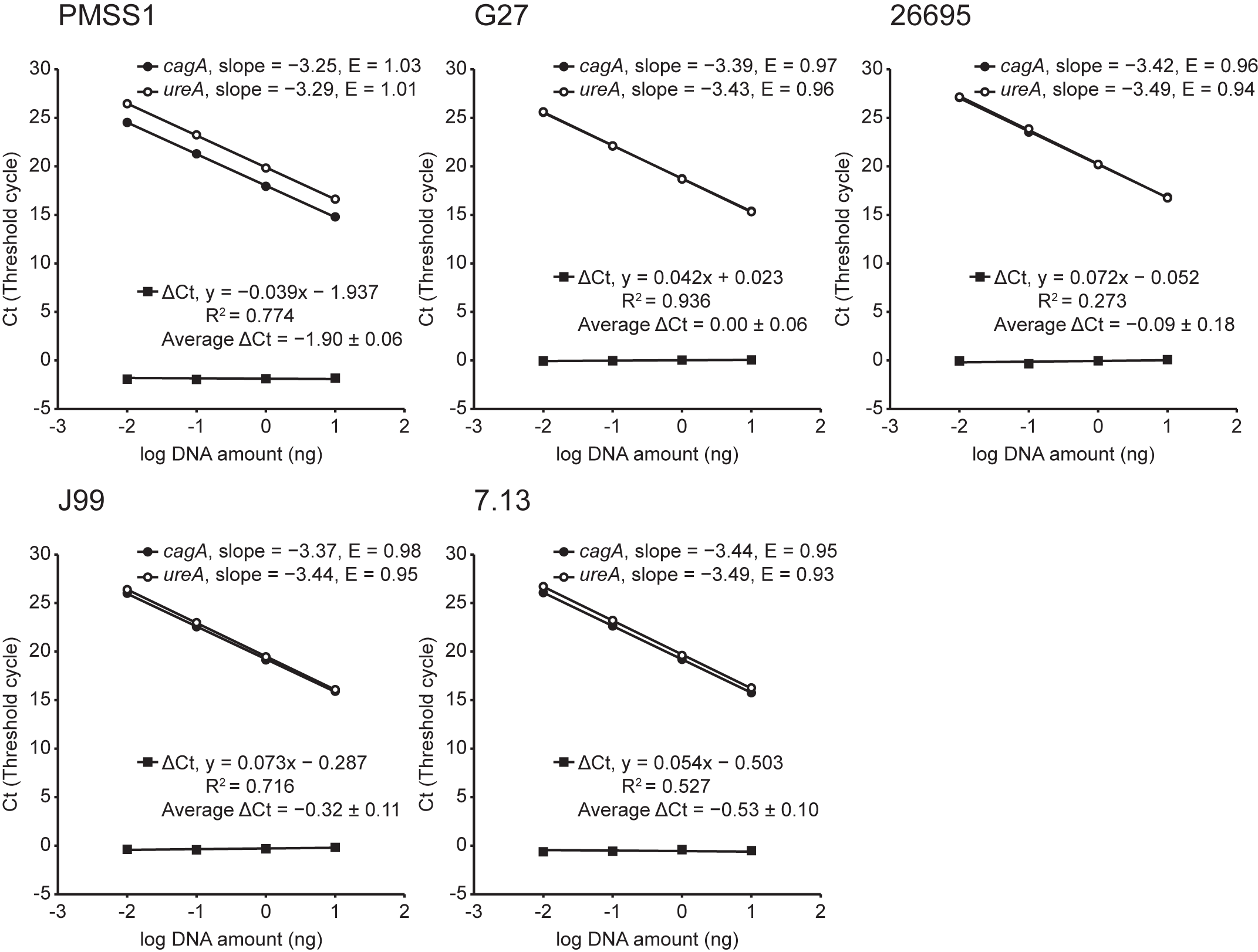

Supplement: Figure S1 [file mbo006163125sf1.tif]

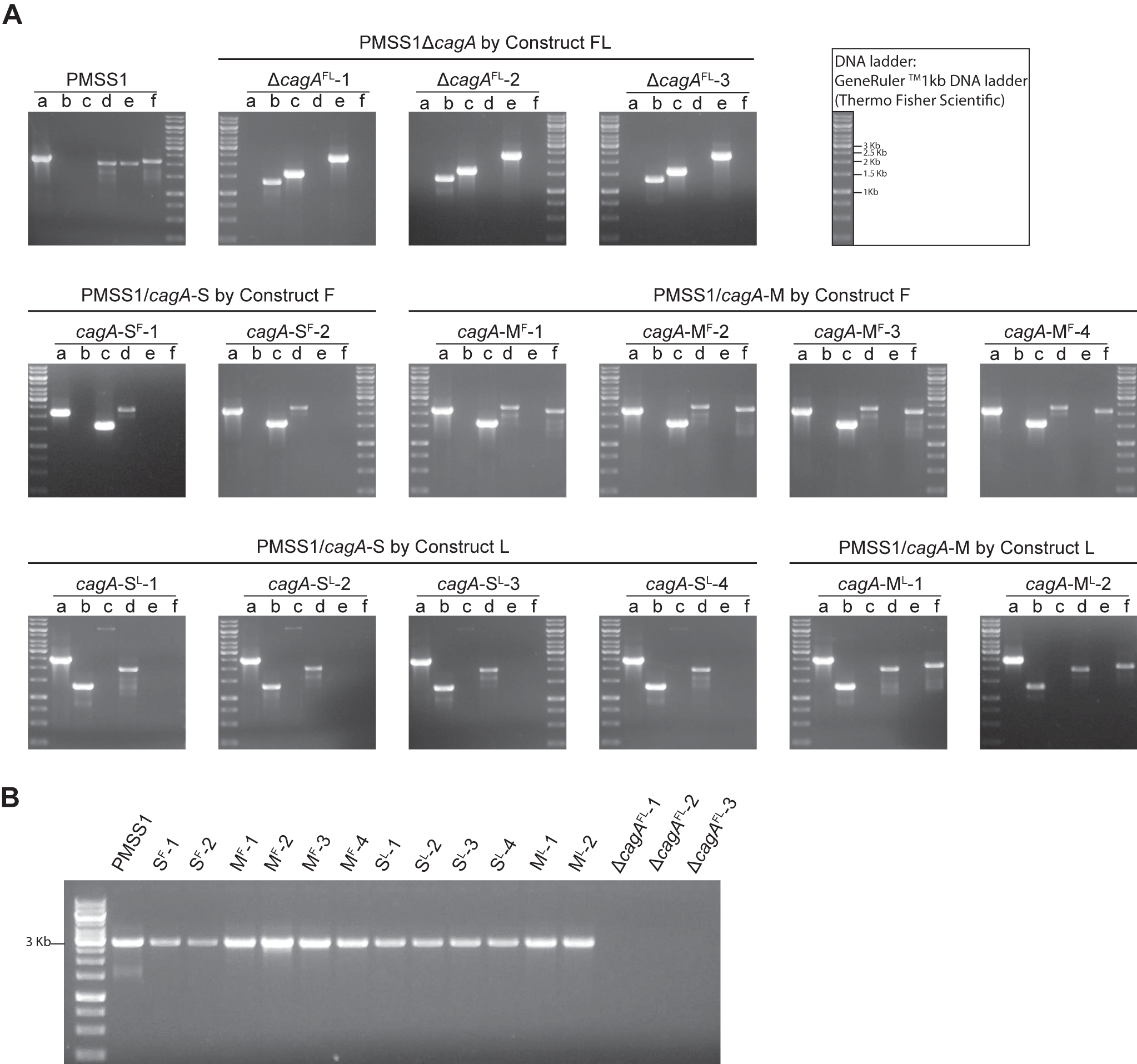

Supplement: Figure S2 [file mbo006163125sf2.tif]

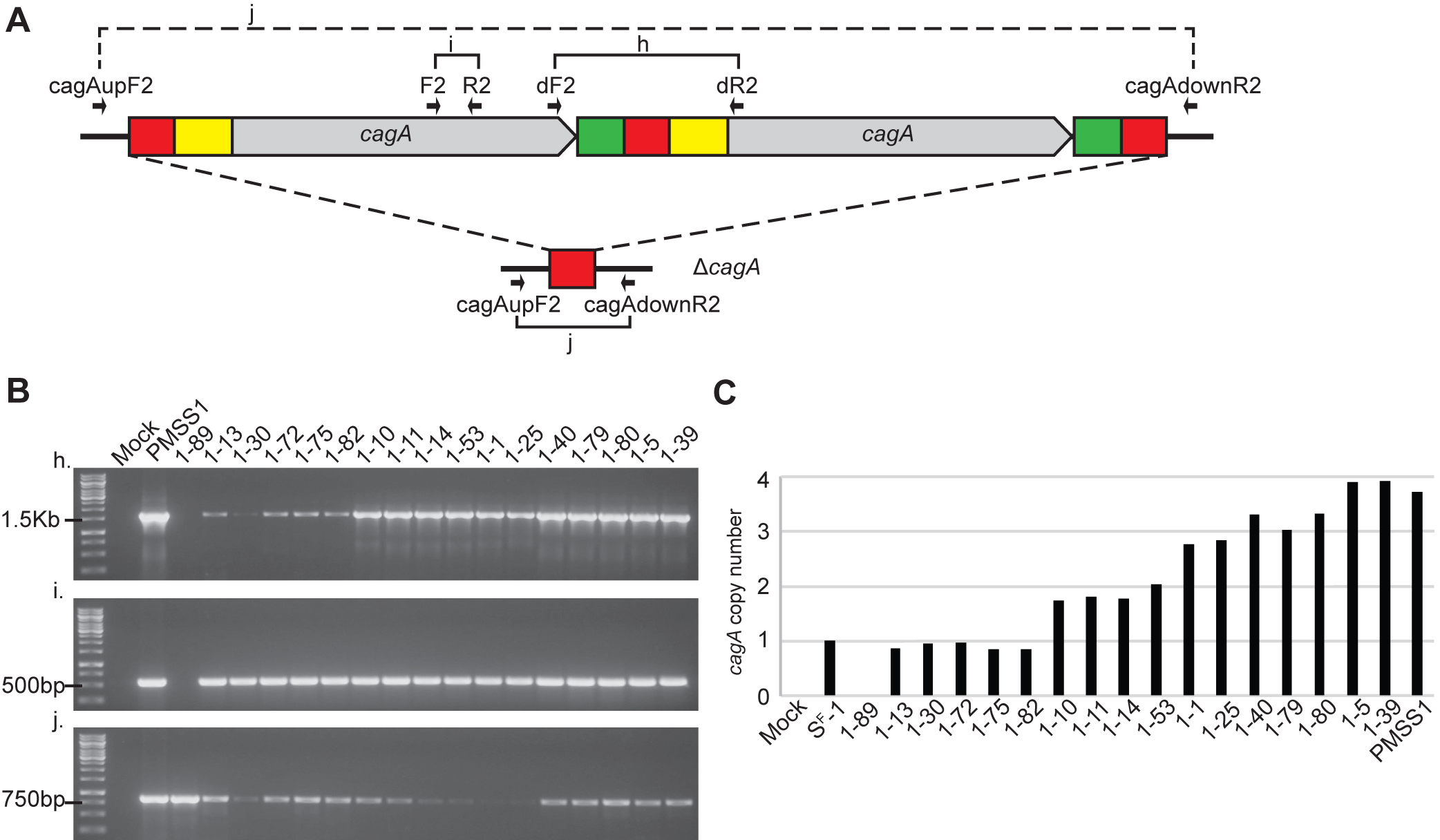

Supplement: Figure S3 [file mbo006163125sf3.tif]

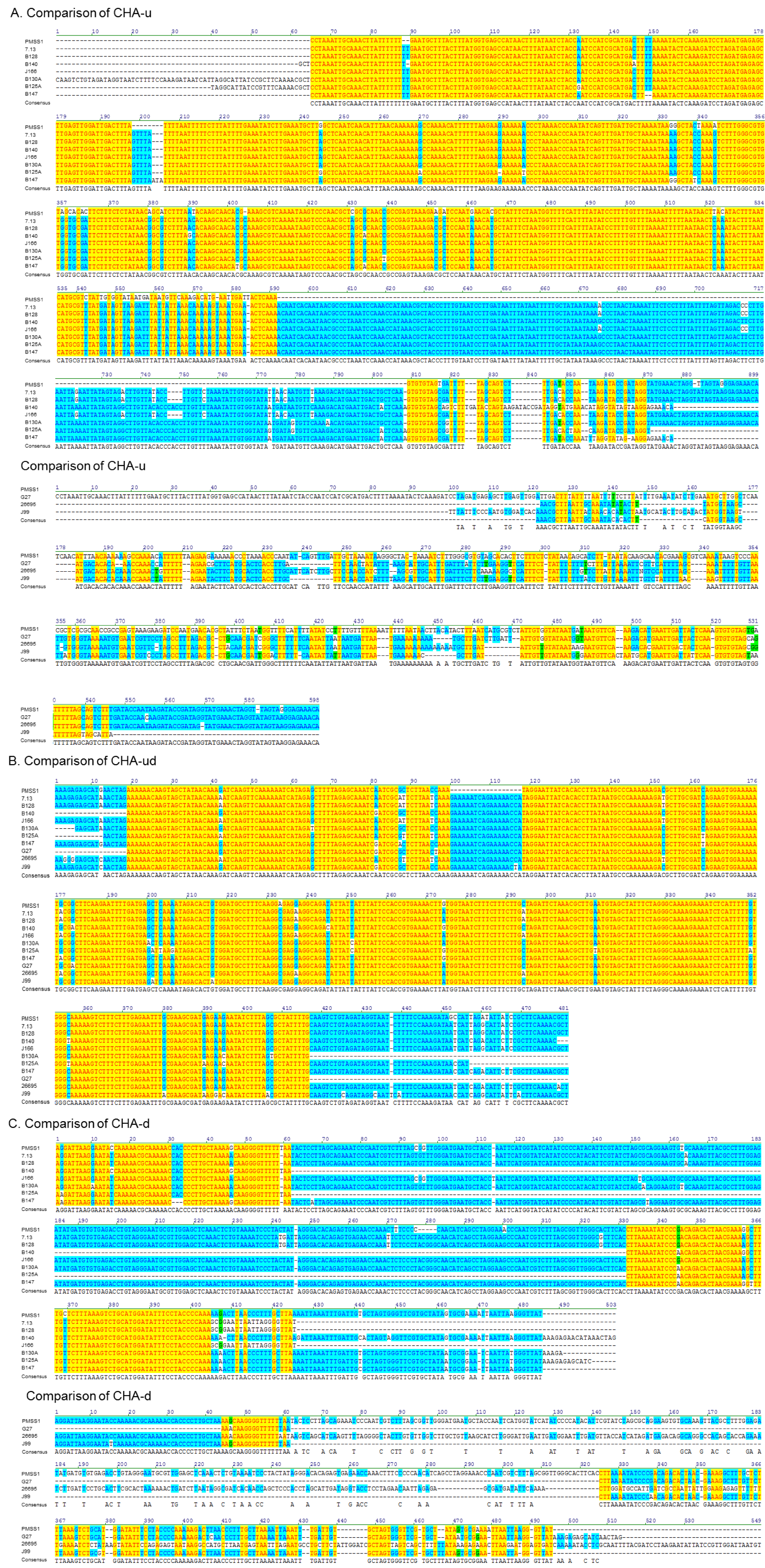

Supplement: Figure S4 [file mbo006163125sf4.tif]
